# Effects of Different Neck Manual Lymphatic Drainage Maneuvers on the Nervous, Cardiovascular, Respiratory and Musculoskeletal Systems in Healthy Students

**DOI:** 10.3390/jcm9124062

**Published:** 2020-12-16

**Authors:** Ángela Río-González, Ester Cerezo-Téllez, Cristina Gala-Guirao, Laura González-Fernández, Raquel Díaz-Meco Conde, Mónica de la Cueva-Reguera, Carlos Guitérrez-Ortega

**Affiliations:** 1 Faculty of Sport Sciences, Universidad Europea de Madrid, Villaviciosa de Odón, 28670 Madrid, Spain; Angela.rio@universidadeuropea.es (Á.R.-G.); cristina.gala@universidadeuropea.es (C.G.-G.); laura.gonzalez@universidadeuropea.es (L.G.-F.); Raquel.diazmeco@universidadeuropea.es (R.D.-M.C.); monica.delacueva@universidadeuropea.es (M.d.l.C.-R.); 2 Sanamanzana Physiotherapy Clinic, 28003 Madrid, Spain; 3 Faculty of Physical Therapy, Universidad de Alcalá, Alcalá de Henares, 28801 Madrid, Spain; 4 Preventive Medicine Department, Hospital Central de la Defensa Gómez Ulla, 28047 Madrid, Spain; cgutort@oc.mde.es

**Keywords:** manual lymphatic drainage, physical therapy, cardiovascular system, musculoskeletal system

## Abstract

The aim of this study is to describe the short-term effects of manual lymph drainage (MLD) isolated in supraclavicular area in healthy subjects. A 4-week cross-sectional, double-blinded randomized clinical trial was conducted. Participants: 24 healthy participants between 18 and 30 years old were recruited from Universidad Europea de Madrid from December 2018 to September 2019. A total of four groups were studied: control, placebo, Vodder, and Godoy. The order of the interventions was randomized. Resting Heart Rate and Oxygen Saturation, blood pressure, pressure pain threshold of trapezius muscle, respiratory rate, range of active cervical movements were measured before and after every intervention. All the participants fulfilled four different interventions with a one-week-wash-out period. No statistically significant differences were found between groups in descriptive data; neither in saturation of oxygen, diastolic blood pressure and cervical range of motion. Significant differences were found in favor of Vodder (*p* = 0.026) in heart rate diminution and in cardiac-rate-reduction. A significant difference in respiratory rate diminution is found in favor of the Godoy group in comparison with the control group (*p* = 0.020). A significant difference is found in favor of the Godoy group in systolic blood pressure decrease (*p* = 0.015) even in pressure pain threshold (*p* < 0.05). MLD decreases systolic blood pressure in healthy participants. However, it does not produce any changes in other physiologic outcomes maintaining physiologic values, which may suggest the safety of the technique in patients suffering from other pathologies.

## 1. Introduction

Manual lymphatic drainage (MLD) is a very light, rhythmic and superficial massage technique focused on increasing the activity of the lymphatic system, as well as reducing the consistency of the edema and its volume using manual techniques on the skin [1,2]. MLD includes maneuvers based on circular or elliptical movements, which first tighten the skin and then relax the contact until the skin returns to the initial position [3]. Some authors have described specific maneuvers in the supraclavicular area (terminus) used in the cervical region as indispensable for the MLD treatment [3,4]. These manual techniques are associated with the specific treatment of the edematized zone and are described to be good for the treatment of patients suffering from edema [4].

MLD techniques have been described as effective over edema-volume and pain reduction [4,5,6], lymphedema prevention [7,8] and on improving pain, sedation and patient well-being. Godoy’s method, which is considered a MLD technique, has also described an amelioration in reducing congenital lymphoedema in pediatric patients using the cervical stimulus as monotherapy [4,9]. Thereby, MLD may help breast cancer and neoplasms patients in their treatment of scars and in improving the range of motion when it is accompanied by other treatments [7].

The autonomic nervous system maintains homeostasis through the coordinated action of sympathetic and parasympathetic systems [10]. In the regulation of respiratory function, the parasympathetic system is the main modulator, performing a bronchoconstrictive action in the smooth muscle [10,11,12]. According to Godoy and Vodder methods, MLD techniques may produce effects on the neurovegetative nervous system, decreasing sympathetic activity and, indirectly, increasing the parasympathetic system and a presumed mechanism of action of stimulating contractions of the lymphangions [13,14,15]; even effects of Vodder maneuvers on intracranial pressures have been described [16]. Godoy maneuvers slide over the skin while Vodder maneuvers only produce a traction of the skin. MLD techniques described in the different methods describe changes in vital signs as result of variations in the autonomic nervous system; the smooth and rhythmic manual technique would inhibit the sympathetic nervous system.

Different methods of MLD techniques, as superficial, slow and rhythmic techniques, described changes on the cardiac and respiratory parameters (heart rate, respiratory rate [17], oxygen saturation, blood pressure), the hemodynamic function (lymphatic and arterial vasoconstriction) [18,19,20], pain level [21] and joint range of movement [15,22], as result of variations in the autonomic nervous system, inhibiting the sympathetic nervous system. In addition, this hypothetical relaxing effect [3,23,24,25,26] suggests that MLD could be beneficial for other types of pathologies related to autonomous disturbance (physiological stress, cardiorespiratory pathology, arterial hypertension, muscular and connective tissue tension, and neurovegetative imbalance). These stimuli have been used in adults in association with the manual and mechanical lymphatic drainage techniques developed in previous investigation [4,13]. However, description of the effects of MLD on basal physiologic parameters in healthy subjects, developed description of the techniques in the methodology or comparison with placebo or control groups, have not been documented yet. Therefore, the aim of this study is to describe the short-term effects of manual lymph drainage (MLD) isolated in supraclavicular area in healthy subjects.

## 2. Experimental Section

### 2.1. Design

A 4-week cross-sectional randomized, blinded, clinical essay was performed to describe the results of different MLD techniques (Vodder and Godoy) on the nervous, cardiovascular, respiratory and musculoskeletal systems compared with a placebo maneuver and control group. Concealed allocation was achieved by having randomization performed by an external researcher, using a closed envelope given to each participant; nobody could see it but the interventor.

A total of four groups were established. One control group and three groups of intervention were defined: Godoy’s MLD, Vodder MLD and placebo. Each participant was randomly assigned to one of the four groups. As a cross over design, each participant served as its own control. The participant did not know the group assignment, nor the therapy received each day; the blinded evaluator did not know about the intervention performed on the participant. The order of the four interventions was randomized, considering a 1-week washout period in between. Each participant’s allocation was placed in a sealed-numbered envelope that was kept at an off-site location. After a participant had been randomized, an independent coordinator gave the therapist the envelope which contained the blinded information about the group allocation and the order of the interventions.

### 2.2. Sample

Of 30 initial candidates, 24 of them meet the inclusion criteria and do not meet the exclusion criteria. A total of 24 healthy students (21.71 ± 3.53 years; 22.26 ± 2.41 BMI) were recruited and followed up on the four interventions programmed by protocol from December 2018 to September 2019. Participants were eligible for the study if they (a) were healthy participants between 18 and 30 years old (b) physiotherapy students, (c) who had never studied or been treated with MLD and (d) who agreed to participate voluntarily and signed the informed consent. Exclusion criteria were: (a) neurological diseases, (b) cardiovascular system diseases, (c) respiratory diseases, (d) systemic diseases (e) infection, (f) trauma history or chronic pain, (g) unstable medical or psychiatric illness or medication use, (h) subjects who use drugs in the last 6 months.

All the participants were asked not to perform intense physical exercise 24 h before the intervention, as well as not to take caffeine, or smoke at least 30 min before their participation (intervention or measures). Environmental conditions such as temperature or visual and acoustic stimuli were maintained constant during the treatments [27]. The intervention was performed by the same therapist and the participants were evaluated by the same blinded evaluator.

### 2.3. Procedures

The study was conducted in accordance with the rules of the Declaration of Helsinki. The protocol was approved by the Commission of Research of Health Sciences of the European University of Madrid CIPI/029/17 and Research Ethics Committee of the Prince of Asturias University Hospital OE 31/2018, and it was also registered at ClinicalTrials.gov ID: NCT03709407.

All the variables were collected before and after each intervention according to the following protocol. All measuring devices were calibrated and validated previously.

(1) Initial assessment (Evaluation V0):

The participant was lying in the supine position for 10 min [28]. After that, the resting heart rate and Oxigen saturation were measured 3 times using the pulse oximeter (Quirumed CMS50D, London, UK) during 3 min [29]. Then, the respiratory rate (RR) was measured by recording the respiratory cycles for 30 s without the participant knowing it, to avoid conditioning [17].

Subsequently, the participants seats relaxed in a chair, with his feet on the floor parallel and his back supported [30]. Blood pressure (BP) (with digital blood pressure monitor: OMRON, M3 (OMRON Healthcare Co. Ltd., Tokyo, Japan) was recorded twice, separated by 1 min, and the average was calculated [31,32]. Then the active cervical range of motion (CROM) was measured by the cervical goniometer (CROM: Performance Attainment Associates, Roseville, The Netherlands) for three times flexion-extension, rotation, and inclination [33]. Each movement was recorded three consecutive times, and after ruling out the smallest of the measurements, the average was calculated [34]. Additionally, in sitting position, the pressure pain threshold was measured at the level of the upper trapezius fibers using an analog algometer (Wagner Instruments, Greenwich, CT, USA) [35,36] at the area of the trapezius trigger point 2, calculating the midpoint of the line drawn between the ipsilateral edge of the spinous process of C5 and the external and posterior edge of the homolateral acromion three times separated by 30 s; the highest measure was discarded and the average was made between the other two [35]. The reproducibility and validity of pressure pain threshold (PPT) have been documented in previous studies [35,36,37,38].

**Figure 1:**
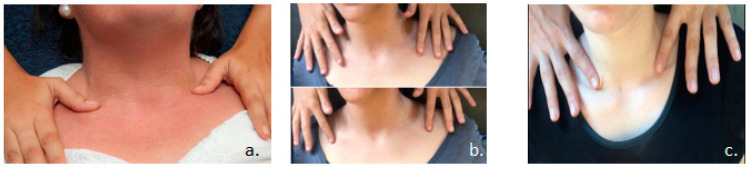
(**a**) Dr. Godoy maneuver, cervical stimuli, (**b**) Terminus Dr. Vodder, (**c**) Placebo maneuver.

(2) Treatment Procedures:

A trained physiotherapist, (10 years’ experience in MLD technique and as a university professor) used specialized hand movements in a range of different sequences at 20–30 stimuli per min for 15 min. The maneuvers were performed as very light, completely pain-free, rhythmical translational movements of the skin in the same direction from lateral to medial close to the supraclavicular fossa. The participants were lying in the supine position and the physiotherapist at the head of the treatment table. In order to maintain consistency, different maneuvers procedure were applied in the same standardized protocols (Figure 1), using the technique (V) described by Dr. Vodder, (G) described by Dr. Godoy, (P) placebo maneuver (superficial sliding on the inferior border of the clavicle, not approaching the supraclavicular fossa, without tensing the skin, making a very smooth glide to middle–lateral direction), or (C) control phase/group (participants did not receive any touch, only lying). The sequence of the four interventions was randomized with a 1-week washout period in between.

(3) Post-intervention assessment-evaluation:

After the corresponding intervention was completed, the participant remained in a supine position to measure the post-intervention variables, all except active CROM, BP and PPT that were performed afterwards in the sitting position as described in the initial assessment. The previous parameter assessment protocol was followed at this moment. When the measurements were completed, the participants were told to carry out their normal daily activity. However, they were informed of the possible side effects of the technique during 30 min after the intervention (hypotension, sensation of relaxation or sleep or mild dizziness). Finally, participants were asked about the appearance of symptoms during a period of 30 min post-intervention.

### 2.4. Data Analysis

Quantitative variables are shown by median (Md) and interquartile range (IQR) when non-parametric condition is defined, otherwise they are shown by mean (M) and standard deviation (SD). The mean values and frequencies of the parameters were assessed by descriptive statistics. For comparing groups, the Mann–Whitney U test was used. To analyze the differences before and after treatment for each group, the Wilcoxon test was used. A *p* < 0.05 was accepted to be statistically significance level. All the data were analyzed using IBM SPSS 24.0 statistical package for Windows (IBM, Chicago, IL, USA).

## 3. Results

Overall, no differences are found among all the groups after each intervention (C, P, V & G) in saturation of oxygen, diastolic blood pressure and CROM, neither on age nor sex (Table 1). Statistically significant difference is found in favor to Vodder group in comparison with placebo group in heart rate diminution reduction (*p* = 0.026). A 5-point-cardiac-rate-reduction is found in Vodder group. A statistically significant difference in respiratory rate diminution is found in favor to Godoy group in comparison with control group (*p* = 0.020).

Vodder group shows a significant increase in systolic blood pressure (*p* = 0.007) in comparison with all groups (C, P, G). A statistically significant difference is found in favor to Godoy group in systolic blood pressure decrease in comparison with Vodder group (*p* = 0.015).

No statistically significant differences are found between groups in CROM on flex-extension, side bending or rotation movements. However, a statistically significant difference was found between the control group and the Godoy group (*p* = 0.024): the control group reduces significantly the side bending cervical range of movement. In addition, a statistically significant difference is found in favor of the Godoy group in comparison with placebo group (*p* = 0.053) in PPT.

## 4. Discussion

The aim of this study is to know if MLD techniques produce effects in neurophysiologic parameters in healthy participants. The majority of these outcomes do not change with the manoeuvers performed, in addition, all statistically significant differences found were included into physiologic parameters. Furthermore, no adverse effects have been described during the execution of the study. This fact shows the safety for its use in pathological participants.

According to Junior et al. [39] the physiological adaptations promoted by different massage maneuvers can be monitored through autonomic nervous system and cardiovascular responses [38]. It would support the results obtained in our manuscript in relation to heart rate, respiratory frequency and oxygen saturation.

Shortage of significant changes may be justified by carrying out the study in healthy participants. In this profile, central and peripheral neuromodulator mechanisms of the Autonomic Nervous System are many and complex, and rapid adjustments are made to the possible modifications that could be caused by the technique studied and in the run-time analyzed [12]. Therefore, future studies are needed to assess the usefulness of these techniques in healthy participants by measuring neurohumeral direct outcomes; as in participants with pathology, where these neuromodulator mechanisms could be exhausted. Aparecida [40], conducted on a similar population (healthy university participants) were intervened with manual therapy maneuvers; however, they measured after a physiological stress situation, reporting an increase in parasympathetic tone [40].

MLD provokes effects over neurovegetative functions such as reducing blood pressure and relaxing the heart muscle in healthy participants [14,22] what agrees with our results. The fact that a statistically significant heart rate decrease occurs in the Autonomous Nervous System in the Vodder group compared to placebo, confirms the fact that the specific physical stimuli provoked by MLD maneuvers can induce relaxation through the stabilization of the autonomic system always included in a security range, not presenting clinical relevance in healthy participants. It also agrees with Lee et al. [41], who showed that physical stimuli can reduce the heart rate during experimental measurements [41]. In the present study, we used MLD maneuvers with a minimum pressure on the skin, in order to check if the parasympathetic effect is really achieved [42]. It agrees with Kim [22], as being an effective technique in reducing the activity of the sympathetic nervous system in healthy participants and suggests that it could help to reduce physiological stress and improve autonomic function by increasing parasympathetic activity [22,43]. This explanation would also justify the statistically significative diminution in respiratory rate observed in Godoy’s group; the fact that a control and placebo group were performed discard any possibility of obtaining changes due to the lain position or patient relaxation. Other authors [42] have also described the effects of therapeutic massage, concluding that it can produce a modification of the activity of the vegetative nervous system, displacing the neurovegetative balance towards a parasympathetic predominance.

Studies have shown the effects of MLD in patients with heart failure [44]. The heart rate also decreased following 15 min of MLD in contrast with all other hemodynamic parameters that were not affected by MLD as in our study. Other authors describe that to achieve effects on the neurovegetative system 15 min are required [45,46,47]. The MLD caused a decrease in heart rate according to Kim [48] and Leduc [44], finding significant differences from the beginning of the intervention in both cases. Despite the MLD application protocols being different in each study, their studies support the results of the present study. It confirms the fact that providing a detailed description of the method, regarding speed, pressure and drainage direction as destination, would improve the quality of future MLD research [49]. Due to the mechanical effect and the location of the maneuvers, stimulation of the anterior cervical fascia could also provoke a neurovegetative modulation, similar to the results obtained by Perez [50] with the fascial work in the cervical area (decrease in heart rate and blood pressure). The theory of the facilitation of parasympathetic activity based on the tactile stimuli justify the changes in the autonomous nervous system [51,52]. Another justification to our results may be related to the remote stimulation of the carotid sinus receptors that influence autonomous nervous system, heart rate, blood pressure and respiratory rate as defined by Robles [12].

According to our findings in the PPT, comparing Godoy vs. placebo, we suspect that the traction and the sliding of the skin of the fossa during the maneuver could both induce changes in the connective tissue around. In the same way as cervical range of movement values in side-bending, reduction may be due to the lie down static position maintained during 15 min without any stimuli.

One of the goals of this study is the cross over design conducted over 4 weeks at approximately the same time of the day to control for the effect of the circadian rhythm on the autonomic nervous system. In addition, considering that MLD effects remains for 24–48 h, a 1-week wash-out period was established. Furthermore, having both a placebo and a control group supports the validity of the results obtained. Our study is the first clinical essay evaluating the systemic effect of the different MLD maneuvers at the supraclavicular fossa.

One of the weaknesses might be the heart rate measurement, instead of heart rate variability (HRV). There is controversy in the evaluation of this value, depending on the basal state of the participant (its initial level of autonomic nervous system (parasympathetic) and the intensity of the stimulation) referred by some authors [53,54], might be a weakness of our study. Other authors refer that heart rate variability is considered a precise, noninvasive, and convenient procedure to investigate the status of the autonomic nervous system and/or target function impairment [55]; however, to avoid this weakness, we assured that the stimuli was exactly the same in all the participants and all of them presented a relaxed status before starting the maneuver.

As a limitation, the sample size makes us consider these results with caution and should be confirmed by further investigations with direct physiologic measurements, in pathologic subjects to value the effect and security of the techniques. Even the composition of the sample, healthy university students, could make a rapid compensation of the changes produced, so it makes it difficult to assess the effect of the technique in other participants. Even the basal parameters, as they were non-pathological, were not susceptible to relevant modifications.

## 5. Conclusions

Our study opens new researching lines for future projects even in pathologies where an autonomic nervous system implication exists, as these techniques are considered safe also in non-healthy subjects. In addition, the fact that these techniques are safe, makes physical therapists confident with them, and opens future research lines. MLD decreases systolic blood pressure in healthy participants. However, it does not produce any changes in other physiologic outcomes, such as cardiovascular or respiratory, which may suggest the safety of the technique in patients suffering from other pathologies. No effects were found in musculoskeletal system. Future studies, in a pathologic sample, such as diabetes or Hypertension should be conducted.

## Figures and Tables

**Table 1:** Results of the outcomes expressed in differences of Medians (interquartile range).

Variables	Placebo (P)		Control (C)		Godoy (G)		Vodder (V)		P vs. C *	P vs. G *	P vs. V *	C vs. G *	C vs. V *	G vs. V *
	Md(MAD)	IC95%	Md(MAD)	IC95%	Md(MAD)	IC95%	Md(MAD)	IC95%						
Oxygen Saturation (mmHg%)	0.53 (1.19)	97.03–97.94	0.15 (1.10)	97.08–97.77	0 (0.99)	96.75–97.79	0.33 (2.35)	96.53–97.83	0.547	0.130	0.932	0.455	0.559	0.513
Systolic blood pressure (mmHg)	2.5 (11.57)	113.71–123.95	1 (7.28)	111.01–122.90	0.5 (11.03)	110.85–122.44	4.75 (5.50)	109.41–118.51	0.308	0.506	**0.007** *	0.503	0.055	0.015 *
Diastolic Blood pressure (mmHg)	1.5 (7.854)	65.13–70.90	1.75 (6.30)	64.32–72.21	3.5 (9.38)	65.79–71.99	0.25 (5.26)	63.58–69.37	0.855	0.664	0.563	0.875	0.141	0.229
Heart Rate (beats per minute)	2.5 (6.68)	61.96–71.22	−3.66 (5.55)	59.94–67.41	5.18 (7.53)	60.77–68.73	−5.35 (5.53)	60.17–68.08	0.922	0.076	0.026 *	0.273	0.107	0.871
Respiratory Rate (cycles per minute)	0 (1.68)	7.64–9.19	0 (1.98)	7.75–8.95	1 (1.44)	7.53–8.71	0.5 (1.97)	7.22–8.89	0.334	0.277	0.467	0.020 *	0.274	0.565
CROM Flexoextension (degrees)	0 (8.50)	118.13–133.54	0 (9.02)	119.5–134.45	0 (13.51)	120.43–134.36	2.5 (10.04)	120.43–136.40	0.717	0.845	0.355	0.986	0.518	0.497
CROM Side bending (degrees)	0 (10.05)	85.05–99.39	2.5 (9.08)	82.95–96.63	0 (7.85)	86.52–99.76	0 (10.45)	87.44–98.81	0.363	0.323	0.909	0.024 *	0.198	0.312
CROM Rotation (degrees)	10 (11.07)	133.45–145.71	5 (14.18)	131.85–142.94	5 (9.99)	135.14–145.07	5 (14.73)	131.43–142.07	0.794	0.406	0.361	0.920	0.955	0.987
Pain Pressure Threshold (Kg/cm^2^)	0 (0.64)	3.16–4.29	0.05 (0.67)	3.23–5.03	0.2 (0.55)	3.21–4.30	−0.05 (0.63)	2.82–4.07	0.097	0.053	0.242	0.201	0.223	0.260

Md: Absolut Median; MAD: Median Absolute Deviation AbsCROM: Cervical Range of motion. * *p* value. A *p* < 0.05 was accepted to be statistically significance level (in bold *). Placebo (P); Control (C); Godoy (G); Vodder (V).

## References

[1] Stuiver M.M., Tusscher M.R.T., Agasi-Idenburg C.S., Lucas C., Aaronson N.K., Bossuyt P.M.M. (2015). Conservative interventions for preventing clinically detectable upper-limb lymphoedema in patients who are at risk of developing lymphoedema after breast cancer therapy. Cochrane Database Syst. Rev..

[2] Ferrandez J.-C. (2006). El Sistema Linfático: Historia, Iconografía e Implicaciones Fisioterapéuticas.

[3] Wittlinger H., Wittlinger D., Wittlinger A.W.M. (2012). Drenaje Manual Según El Método Del Dr. Vodder.

[4] De Godoy J.M.P., Meza M.C. (2008). Godoy & Godoy technique of cervical stimulation in the reduction of edema of the face after cancer treatment. QJM Int. J. Med..

[5] Kim S.-J., Yi C.-H., Kwon O.-Y. (2007). Effect of complex decongestive therapy on edema and the quality of life in breast cancer patients with unilateral lymphedema. Lymphology.

[6] Vairo G.L., Miller S.J., Rier N.M.C.I., Uckley W.B.I. (2009). Systematic Review of Efficacy for Manual Lymphatic Drainage Techniques in Sports Medicine and Rehabilitation: An Evidence-Based Practice Approach. J. Man. Manip. Ther..

[7] Lacomba M.T., Sánchez M.J.Y., Goñi Á.Z., Merino D.P., Del Moral O.M., Téllez E.C., Mogollón E.M. (2010). Effectiveness of early physiotherapy to prevent lymphoedema after surgery for breast cancer: Randomised, single blinded, clinical trial. BMJ.

[8] Castro-Sánchez A.M., Moreno-Lorenzo C., Matarán-Peñarrocha G.-A., Aguilar-Ferrándiz M.E., Almagro-Céspedes I., Anaya-Ojeda J. (2011). Prevención del linfedema tras cirugía de cáncer de mama mediante ortesis elástica de contención y drenaje linfático manual: Ensayo clínico aleatorizado. Med. Clín..

[9] De Godoy J.M.P., De Godoy A.C.P., Guimarães T.D., Godoy M.D.F.G.D. (2012). The Godoy & Godoy Cervical Stimulation Technique in The Treatment of Primary Congenital Lymphedema. Pediatr. Rep..

[10] Cardinali D.P. (2018). Autonomic Nervous System: Basic and Clinical Aspects.

[11] Idiaquez J., Benarroch E.E., Nogues M. Evaluation and Management of Autonomic Disorders: A Case-Based Practical Guide. https://descubre-uem.bibliocrai.universidadeuropea.es/cgi-bin/koha/opac-detail.pl?biblionumber.

[12] Cabrera A.R., Chávez A.M., Rojas R.C.C., Kessler C.M., Delgado G., Vidal B.E. (2014). Los barorreflejos arteriales cardiovagal, cardiosimpático y vasosimpático y el control neural de la presión arterial a corto plazo. Rev. Neurol..

[13] De Godoy J.M.P. (2010). Godoy & Godoy technique in the treatment of lymphedema for under-privileged populations. Int. J. Med. Sci..

[14] Wittlinger G., Wittlinger H., Harris R.H. (2019). Vodder’s Manual Lymph Drainage: A Practical Guide.

[15] Hutzschenreuter P., Ehlers R. (1986). Effect of manual lymph drainage on the autonomic nervous system. Z. Lymphologie. J. Lymphology.

[16] Roth C., Stitz H., Ferbert A., Deinsberger W., Pahl R., Engel H., Kleffmann J. (2016). Craniocervical manual lymphatic drainage and its impact on intracranial pressure—A pilot study. Eur. J. Neurol..

[17] Potter P.A., Perry A.G., Stockert P., Hall A. (2013). PSES—Fundamentals of Nursing.

[18] Chikly B.J. (2005). Manual techniques addressing the lymphatic system: Origins and development. J. Am. Osteopat. Assoc..

[19] Kasseroller R.G. (1998). The vodder school: The vodder method. Cancer.

[20] Horst W., Schuchhardt C. (2008). Lymphedema: Diagnosis and Therapy.

[21] Ekici G., Bakar Y., Akbayrak T., Yuksel I. (2009). Comparison of Manual Lymph Drainage Therapy and Connective Tissue Massage in Women with Fibromyalgia: A Randomized Controlled Trial. J. Manip. Physiol. Ther..

[22] Kim S.-J., Kwon O., Yi C. (2009). Effects of manual lymph drainage on cardiac autonomic tone in healthy subjects. Int. J. Neurosci..

[23] Torres-Lacomba M., Salvat Salvat I. (2006). Guía de Masoterapia Para Fisioterapeutas.

[24] Gallego J.V. (2009). Manual Profesional Del Masaje. Guía Práctica.

[25] Tortora G.J., Derrickson B. (2018). Principios de Anatomía y Fisiología.

[26] Shim J.-M., Kim S.-J. (2014). Effects of Manual Lymph Drainage of the Neck on EEG in Subjects with Psychological Stress. J. Phys. Ther. Sci..

[27] Wilmore J.H., Costill D.L. (2004). Fisiología del Esfuerzo y del Deporte.

[28] Yamaya Y., Bogaard H.J., Wagner P.D., Niizeki K., Hopkins S.R. (2002). Validity of pulse oximetry during maximal exercise in normoxia, hypoxia, and hyperoxia. J. Appl. Physiol..

[29] Stieb D.M., Shutt R., Kauri L., Mason S., Chen L., Szyszkowicz M., Dobbin N.A., Rigden M., Jovic B., Mulholland M. (2017). Cardio-Respiratory Effects of Air Pollution in a Panel Study of Outdoor Physical Activity and Health in Rural Older Adults. J. Occup. Environ. Med..

[30] Pinar R., Sabuncu N., Oksay A. (2004). Effects of crossed leg on blood pressure. Blood Press..

[31] Carey R.M., Whelton P.K. (2018). Prevention, Detection, Evaluation, and Management of High Blood Pressure in Adults: Synopsis of the 2017 American College of Cardiology/American Heart Association Hypertension Guideline. Ann. Intern. Med..

[32] Gómez Marcos M.Á., García Ortiz L., Sánchez Rodríguez Á., Parra Sánchez J., García García Á., González Elena L.J. Control de La Presión Arterial, Concordancias y Discrepancias Entre Diferentes Métodos de Medida Utilizados. http://www.elsevier.es.

[33] Youdas J.W., Carey J.R., Garrett T.R. (1991). Reliability of Measurements of Cervical Spine Range of Motion—Comparison of Three Methods. Phys. Ther..

[34] Audette I., Dumas J.-P., Côté J.N., De Serres S.J. (2010). Validity and Between-Day Reliability of the Cervical Range of Motion (CROM) Device. J. Orthop. Sports Phys. Ther..

[35] Fischer A.A. (1998). Algometry in Diagnosis of Musculoskeletal Pain and Evaluation of Treatment Outcome: An Update. J. Musculoskelet. Pain.

[36] Persson A.L., Brogårdh C., Sjölund B.H. (2004). Tender or not tender: Test-retest repeatability of pressure pain thresholds in the trapezius and deltoid muscles of healthy women. J. Rehabil. Med..

[37] Edwards J., Knowles N. (2003). Superficial Dry Needling and Active Stretching in the Treatment of Myofascial Pain—A Randomised Controlled Trial. Acupunct. Med..

[38] Cerezo-Téllez E., Torres-Lacomba M., Fuentes-Gallardo I., Perez-Muñoz M., Mayoral-Del-Moral O., Lluch-Girbés E., Prieto-Valiente L., Falla D. (2016). Effectiveness of Dry Needling for Chronic Nonspecific Neck Pain: A Randomized, Single-Blinded, Clinical Trial. Pain.

[39] Junior N.M., Junior E.P., Siqueira M., Cavina A.P.D.S., Pastre C.M., Vanderlei F.M. (2018). Effects of massage as a recuperative technique on autonomic modulation of heart rate and cardiorespiratory parameters: A study protocol for a randomized clinical trial. Trials.

[40] Aparecida H., Borghi F., Costa G.T., Bortz A.V., Da Silva P.C., Grassi-Kassisse D.M. (2017). Manual therapy program induced alterations in autonomic nervous system modulation of students preparing to apply for the universities: One-group, pre- and post-test study. Curr. Res. Integr. Med..

[41] Lee Y.-H., Park B.N.R., Kim S.H. (2011). The Effects of Heat and Massage Application on Autonomic Nervous System. Yonsei Med. J..

[42] Field T. (2016). Massage therapy research review. Complement. Ther. Clin. Pract..

[43] Bender P.U., Da Luz C.M., Feldkircher J.M., Nunes G.S. (2019). Massage therapy slightly decreased pain intensity after habitual running, but had no effect on fatigue, mood or physical performance: A randomised trial. J. Physiother..

[44] LeDuc O., Crasset V., Leleu C., Baptiste N., Koziel A., Delahaie C., Pastouret F., Wilputte F., LeDuc A. (2011). Impact of manual lymphatic drainage on hemodynamic parameters in patients with heart failure and lower limb edema. Lymphology.

[45] Moyer C.A., Rounds J., Hannum J.W. (2004). A Meta-Analysis of Massage Therapy Research. Psychol. Bull..

[46] Diego M.A., Field T., Sanders C., Hernandez-Reif M. (2004). Massage therapy of moderate and light pressure and vibrator effects on EEG and heart rate. Int. J. Neurosci..

[47] Caufriez M., Fernandezdominguez J.C., Perinel P., Mairlot S., Dierick F., Van Ostaeyen B. (2011). Contribución al estudio de los efectos sistémicos neurovegetativos del masaje terapéutico mediante el análisis espectral de la variabilidad de la frecuencia cardiaca. Rev. Iberoam. Fisioter. Kinesiol..

[48] Lee M.S., Kim H.-J., Song J., Park K.-W., Moon S.-R. (2004). Effects of multifunctional fabrics on cardiac autonomic tone and psychological state. Int. J. Neurosci..

[49] Thompson B., Gaitatzis K., De Jonge X.J., Blackwell R., Koelmeyer L.A. (2020). Manual lymphatic drainage treatment for lymphedema: A systematic review of the literature. J. Cancer Surviv..

[50] Fernández-Pérez A.M. (2011). Efectos Neurofisiológicos, Psicoinmunológicos y Psicológicos a Corto Plazo En Sujetos Sometidos a Técnicas de Inducción Miofascial. http://digibug.ugr.es/handle/10481/20539.

[51] Holey E., Cook E.M. (2012). Evidence-Based Therapeutic Massage E-Book: A Practical Guide for Therapists.

[52] Fernández-Pérez A.M., Peralta-Ramirez M.I., Pilat A., Villaverde C. (2008). Effects of Myofascial Induction Techniques on Physiologic and Psychologic Parameters: A Randomized Controlled Trial. J. Altern. Complement. Med..

[53] Goldberger J.J., Kim Y.-H., Ahmed M.W., Kadish A.H. (1996). Effect of Graded Increases in Parasympathetic Tone on Heart Rate Variability. J. Cardiovasc. Electrophysiol..

[54] Tulppo M.P., Mäkikallio T.H., Seppänen T., Airaksinen J.K.E., Huikuri H.V. (2017). Heart rate dynamics during accentuated sympathovagal interaction. Am. J. Physiol. Circ. Physiol..

[55] Zygmunt A., Stanczyk J. (2010). Methods of evaluation of autonomic nervous system function. Arch. Med. Sci..

